# Pentamidine rescues contractility and rhythmicity in a *Drosophila* model of myotonic dystrophy heart dysfunction

**DOI:** 10.1242/dmm.021428

**Published:** 2015-12-01

**Authors:** Mouli Chakraborty, Estela Selma-Soriano, Emile Magny, Juan Pablo Couso, Manuel Pérez-Alonso, Nicolas Charlet-Berguerand, Ruben Artero, Beatriz Llamusi

**Affiliations:** 1 Translational Genomics Group, Incliva Health Research Institute, Avda. Menendez Pelayo 4 acc 46010, Valencia, Spain; 2 Department of Genetics and Estructura de Recerca Interdisciplinar en Biotecnologia i Biomedicina (ERI BIOTECMED), Universitat de València, Dr Moliner 50, Burjasot 46100, Spain; 3 School of Life Sciences, University of Sussex, Falmer, Brighton, East Sussex, BN1 9QG, UK; 4 Translational Medicine and Neurogenetics, Institut de Génétique et de Biologie Moléculaire et Cellulaire (IGBMC), 1 Rue Laurent Fries, 67400 Illkirch-Graffenstaden, France

**Keywords:** *Drosophila*, Heart dysfunction, Myotonic dystrophy, Muscleblind, Pentamidine

## Abstract

Up to 80% of individuals with myotonic dystrophy type 1 (DM1) will develop cardiac abnormalities at some point during the progression of their disease, the most common of which is heart blockage of varying degrees. Such blockage is characterized by conduction defects and supraventricular and ventricular tachycardia, and carries a high risk of sudden cardiac death. Despite its importance, very few animal model studies have focused on the heart dysfunction in DM1. Here, we describe the characterization of the heart phenotype in a *Drosophila* model expressing pure expanded CUG repeats under the control of the cardiomyocyte-specific driver *GMH5-Gal4*. Morphologically, expression of 250 CUG repeats caused abnormalities in the parallel alignment of the spiral myofibrils in dissected fly hearts, as revealed by phalloidin staining. Moreover, combined immunofluorescence and *in situ* hybridization of Muscleblind and CUG repeats, respectively, confirmed detectable ribonuclear foci and Muscleblind sequestration, characteristic features of DM1, exclusively in flies expressing the expanded CTG repeats. Similarly to what has been reported in humans with DM1, heart-specific expression of toxic RNA resulted in reduced survival, increased arrhythmia, altered diastolic and systolic function, reduced heart tube diameters and reduced contractility in the model flies. As a proof of concept that the fly heart model can be used for *in vivo* testing of promising therapeutic compounds, we fed flies with pentamidine, a compound previously described to improve DM1 phenotypes. Pentamidine not only released Muscleblind from the CUG RNA repeats and reduced ribonuclear formation in the *Drosophila* heart, but also rescued heart arrhythmicity and contractility, and improved fly survival in animals expressing 250 CUG repeats.

## Abstract (precis)

**Summary:** The authors characterize a cardiac-dysfunction model of DM1 in *Drosophila*, and show that pentamidine rescues both contractility and rhythmicity.

## INTRODUCTION

Myotonic dystrophy type 1 (DM1) is the most frequently inherited neuromuscular disease in adults. Aside from skeletal muscle symptoms, multi-organ involvement is also common and typically affects cardiac, endocrine and central nervous system tissues (Thornton, 2014). DM1 [Online Mendelian Inheritance of Man (OMIM) 160900] has been identified as an autosomal-dominant disorder associated with the presence of an abnormal CTG trinucleotide repeat expansion in the 3′ untranslated region (UTR) of the gene encoding myotonic dystrophy protein kinase (*DMPK*) on chromosome 19. Whereas 5-34 CTG repeats are observed in normal alleles, their number can reach up to between 50 and 2000 in DM1 (Brook et al., 1992; Fu et al., 1992; Mahadevan et al., 1992). The best-characterized effect of the expanded *DMPK* RNA (CUG^exp^ RNA) is disruption of the function of RNA-binding proteins, including muscleblind-like 1 (MBNL1) and CUGBP Elav-like family member 1 (CELF1), which regulate multiple RNA-processing events, including alternative splicing, translation, polyadenylation, miRNAs biogenesis, mRNA stability and mRNA intracellular localization (Lee and Cooper, 2009; Batra et al., 2014; Meola et al., 2013; Rau et al., 2011; Adereth et al., 2005; Wang et al., 2012; Wang et al., 2015). CUG^exp^ RNA impairs normal postnatal alternative-splicing transitions regulated by MBNL1 and CELF1. Whereas MBNL1 is sequestered to the CUG repeats, the toxic effect of mutant RNA on CELF1 activity is very complex, and involves increased CELF1 protein levels as a result of its stabilization in the nucleus (Kuyumcu-Martinez et al., 2007; Kim et al., 2014; Timchenko, 2013; Timchenko et al., 2001). As a result of disrupting the function of these proteins, several mis-splicing defects have been described and have been linked to specific symptoms of the disease (Mankodi et al., 2002; Savkur et al., 2001; Tang et al., 2012; Fugier et al., 2011). However, the physiological consequences of alternative splicing, gene expression and microRNA alterations in the heart are yet to be clarified (Phillips et al., 1999; Rau et al., 2011; Kalsotra et al., 2014; Zu et al., 2011; Lopez Castel et al., 2011; Moseley et al., 2006; Perbellini et al., 2011; Fernandez-Costa et al., 2013; Wang et al., 2015). In general, cardiac involvement, which often precedes the skeletal muscle one, occurs in 80% of individuals with DM1 and represents the second most common cause of death of such individuals, after respiratory failure (Vinereanu et al., 2004). Several studies have reported an overall positive association between CTG-repeat size and cardiac involvement, and between the degree of neuromuscular and cardiac dysfunction (Petri et al., 2012; Groh et al., 2002; Dello Russo et al., 2006).

Three interrelated cardiac phenotypes are observed in individuals with DM1. The first is conduction defects, which are particularly common and can progress to complete heart blockage (Nguyen et al., 1988). The second is the development of potentially fatal ventricular and/or atrial arrhythmias (Nigro et al., 2012; Benhayon et al., 2015). The third phenotype, although rarer, is mechanical diastolic and/or systolic dysfunction that can progress to combined systolic and diastolic heart failure (Phillips and Harper, 1997; Mathieu et al., 1999; Lazarus et al., 2002; Groh et al., 2008). The majority of individuals with DM1 show abnormal electrocardiography (ECG) assessments, with prolonged time of conduction of the sinoatrial impulse to the ventricles (PR interval) (20-40% of affected individuals) and ventricular depolarization (QRS complex) widening (5-25%) (McNally and Sparano, 2011). Moreover, echocardiogram studies have also found that some individuals with DM1 have reduced heart contractility, as revealed by a lower left ventricular ejection fraction (LVEF less than 50%) (Dhand et al., 2013; Chaudhry and Frishman, 2012).

**TRANSLATIONAL IMPACT**

**Clinical issue**

Cardiac involvement is a common complication of the skeletal muscle disorder myotonic dystrophy type 1 (DM1), occurring in 80% of DM1 cases. Heart dysfunction is the second most common cause of fatality associated with the disease, after respiratory distress. DM1 is caused by the expansion of an unstable CTG repeat in the 3′ untranslated region (UTR) of the *DMPK* gene, which encodes myotonic dystrophy protein kinase. The expanded CUG repeats form a hairpin that sequesters the RNA-binding protein muscleblind-like 1 (MBNL1) and other nuclear factors into ribonuclear foci in a manner that is proportional to the CUG expansion size. Sequestration has been proposed to cause depletion of these proteins, leading to defects in splicing that underlie some of the clinical symptoms of DM1. Despite the central involvement of heart failure in DM1, very few studies have focused on the molecular cause of cardiac dysfunction in this disease and fewer have tested the effect of potential anti-DM1 compounds on this phenotype using suitable animal models.

**Results**

In this study, Beatriz Llamusi and colleagues generated and characterized a *Drosophila* model expressing pure expanded CUG repeats under the control of the cardiomyocyte-specific driver *GMH5-Gal4*. Supporting the suitability of this model to investigate cardiac dysfunction in DM1, the authors noted key similarities between the cardiac phenotype in DM1 model flies and those documented in individuals with DM1. First, they observed a reduction of median survival in model flies, which correlates to that reported in humans with DM1. Second, they observed a significantly increased heart period and arrhythmia index in the fly model, in line with heart conduction abnormalities that are common in DM1. Thirdly, they observed systolic and diastolic dysfunction reminiscent of that reported in affected humans. DM1 individuals with cardiac abnormalities generally show various extents of heart chamber dilation and hypertrophy, which result in decreased ventricular ejection. This phenotype was also mimicked in the fly model, which demonstrates reduced fractional shortening. Providing proof-of-concept, the authors also reported the efficacy of a known anti-DM1 compound, pentamidine, to partially rescue these heart phenotypes. Adult DM1 model flies fed with pentamidine showed reduced arrhythmicity and improved contractility, allowing a rescue of cardiac output that translated into a median survival that did not differ from control flies expressing 20× CUG repeats. However, the heart-rate dysfunction observed in the DM1 model flies was not completely rescued by pentamidine.

**Implications and future directions**

A better understanding of the molecular mechanisms altered by expansion of CTG repeats, and of the molecular interactions of the repeat sequence *in vivo*, is crucial for deciphering the origin of the symptoms of DM1 and to generate appropriate treatments. This work describes, for the first time, the toxic effects of long CUG RNA on cardiac function in a *Drosophila* model, paving the way for further studies to elucidate the molecular alterations underlying cardiac involvement in DM1. Moreover, the ability to detect changes in the phenotype in response to treatment with a known anti-DM1 compound confirms the specificity of the phenotype and its ability to respond to therapeutic intervention. Because several aspects of DM1 pathogenesis are still unclear, this model could be used to provide a more detailed description of heart involvement in DM1 and allow the identification of potential genetic modifiers of the heart alterations. Importantly, the model can also be used to test the efficacy of different therapeutic approaches that so far have only been tested in skeletal muscle.

Despite the relevance of heart involvement in DM1, the molecular mechanisms causing the abnormalities in electric conduction or contractility are not well understood. In previous inducible DM1 mouse models, conduction disturbances appeared a few days after inducing acute expression of either a short stretch of five CTG triplets or long interrupted CTG repeats (Wang et al., 2007; Mahadevan et al., 2006). These studies pointed to alterations in cardiac conduction and excitability properties as an early event in the appearance of DM1-associated cardiomyopathies. Another mouse model was generated more recently that carries the human DM1 locus constitutively expressed under the regulation of its own promoter and its *cis-*regulatory elements (DMSXL). These mice constitute a good model of slow and steady-state expression of the triplet expansion, as is observed in individuals with DM1. However, cardiac abnormalities (reduced ventricular myocardium cell excitability) were not observed in baseline conditions; rather, they were only revealed after injection of the sodium-channel blocker flecainide (Algalarrondo et al., 2015).

Adult *Drosophila* possess an open circulatory system consisting of a dorsal vessel, which is the 1-mm-long pulsatile heart tube, and an anterior aorta that extends through the thorax and into the head (for a review of *Drosophila* heart development and assessment see Ocorr et al., 2014). The simple structure and physiology of the *Drosophila* heart tube, together with its readily available genetics, provide a suitable *in vivo* assay system for studying cardiac dysfunctions. Here, we report the first *Drosophila* DM1 heart-dysfunction model, generated by overexpression of long pure CUG repeats {250 CUG repeats [CUG(250)×]} under the control of the cardiomyocyte-specific driver *GMH5-Gal4*. We have detected CUG ribonuclear foci and Muscleblind sequestration (the main molecular features of the disease in humans) in the *Drosophila* heart cell nuclei, and a shortened median survival and lifespan in DM1 flies. We also measured several *Drosophila* heart parameters and found that these also resemble the heart dysfunction found in DM1 humans. Importantly, we confirmed that oral administration of pentamidine to flies expressing long CUG repeats releases Muscleblind from these repeats and prevents foci formation in cardiac cell nuclei, also rescuing a subset of heart-dysfunction phenotypes. Our data suggest that *Drosophila* represents an appropriate DM1 heart-dysfunction model for physiopathological studies and supports the utility of this model for the heart-specific testing of potential therapeutic compounds.

## RESULTS

### Generation and characterization of a DM1 heart-dysfunction model in *Drosophila*

To develop a heart-dysfunction model of DM1 in flies, we have generated UAS-CTG transgenic lines carrying 20 [CUG(20)×] or 250 [CUG(250)×] pure CTG repeats and crossed them with the cardiac-specific driver *GMH5-Gal4* (Wessells et al., 2004), which includes the UAS-GFP reporter, allowing the labeling of the tissues in which Gal4 is expressed. The level of expression of the repeats was assessed by qPCR analysis using primers against the common SV40 terminator (Fig. S1), and the transgenes were confirmed to express the expected number of repeats (Fig. S2).

**Fig. 1.**
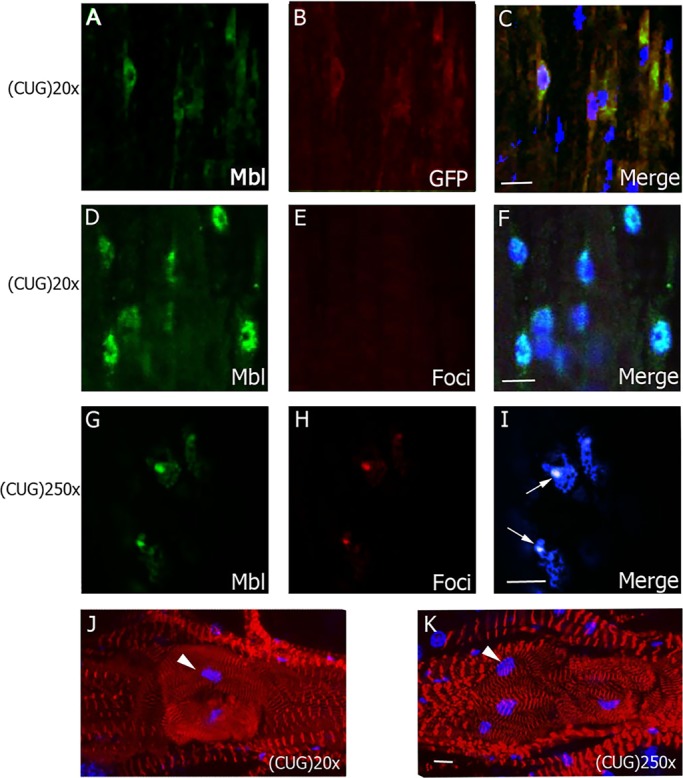
**Characterization of the DM1 heart-dysfunction phenotype in flies.** Representative fluorescent confocal images of adult heart cells from flies expressing short [CUG(20)×] or long [CUG(250)×] repeats under the control of *GMH5-Gal4*. (A-C) In cardiomyocytes expressing CUG(20)×, revealed by anti-GFP antibody (red, B), Muscleblind signal (green, A) was dispersed in the nuclei and cytoplasm. (D-I) Combined immunodetection of Muscleblind (green, D and G) and FISH to detect ribonuclear foci (red, E and H) revealed dispersed expression of Muscleblind and absence of foci in flies expressing short CUG repeats (D-F). However, in flies expressing long CUG repeats (G-I), Muscleblind colocalized with ribonuclear foci (arrows). (J,K) Representative confocal stacks of phalloidin (red)-stained spiral fibers in the region surrounding the ostia in adult hearts (posterior A2-anterior A3 segment) reveals details of the heart structure, in particular increased fiber disorganization in GMH5-Gal4›CUG(250)× flies. Arrowhead points to ostia-associated nuclei. Merged images in C,F,I,J and K include DAPI (blue) counterstaining of the nuclei. All images are from 7-day-old flies. Scale bars: 10 µm.

Hearts of flies expressing CUG repeats under the *GMH5-Gal4* driver were dissected for immunohistological and morphological assessment. Given the crucial involvement of Muscleblind protein in DM1 pathogenesis, it was highly relevant to confirm its expression in the *Drosophila* adult heart. Previous studies of Muscleblind expression in *Drosophila* have focused on adult skeletal muscle (Llamusi et al., 2013) or in the embryo (Artero et al., 1998). In the current study, using an anti-Muscleblind antibody (Houseley et al., 2005), we observed Muscleblind expression in the adult heart cardiomyocytes. Muscleblind displayed a diffuse expression not only in the nucleus but also in the cytoplasm of cardiomyocytes, in both control (OrR) (not shown) and short-repeat-expressing flies (Fig. 1A-F). In contrast, Muscleblind was found concentrated in CUG ribonuclear foci in the nuclei in flies expressing long CUG expansions. Muscleblind sequestration is one of the main features of DM1. Ribonuclear foci were only present in the nuclei of heart cells in long-CUG-expressing flies (Fig. 1G-I).

We also assessed the heart structure by staining actin, a structural component of the contractile machinery of muscles. *Drosophila* heart tubes have two types of muscle fibers, each with distinct myofibrillar structures (Mery et al., 2008; Taghli-Lamallem et al., 2008): (1) spirally or transversely oriented myofibrils that represent the contractile ‘working’ myocardium; and (2) longitudinally oriented myofibrils that are found along the ventral surface of the tube (Molina and Cripps, 2001). In young flies, both types of myofibrils exhibit a tight and well-aligned arrangement. Cardiac myofibrils have been reported to stain uniformly along the entire length of the thin filament with phalloidin (Ao and Lehrer, 1995), so it can be used to visualize both types of myofibrils.

Phalloidin staining of actin did not reveal gross structural abnormalities in the heart tube but found slight differences between flies expressing short or long CUG repeats in the areas surrounding the ostia. Cardiac myofibrils were tightly arranged and well aligned in short-repeat-expressing flies at 1 week of age. However, age-matched flies expressing long CUG repeats in heart showed abnormalities in the parallel alignment of transverse myofibrils, which showed a remarkable spiral disposition and less organized and compact arrangement of the myocardial myofibrils. These alterations have been also reported in aged fly hearts (Paternostro et al., 2001; Wessells et al., 2004; Taghli-Lamallem et al., 2008) (Fig. 1J-K).

### DM1 flies show a median survival reduction and arrhythmicity

**Fig. 2.**
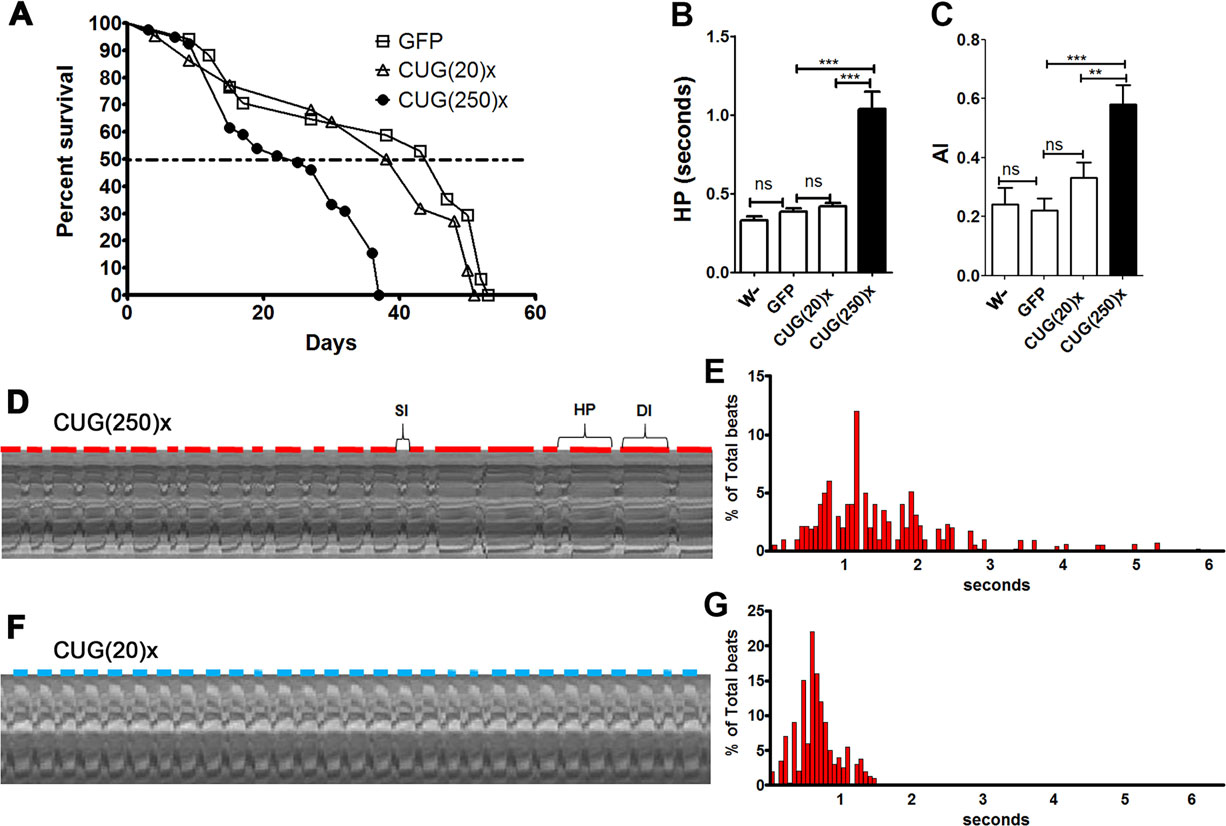
**Flies expressing long CUG repeats in *Drosophila* cardiomyocytes have a shortened median survival and increased arrhythmicity.** (A) Average percentage of live flies, with the genotypes indicated, versus age (in days). Horizontal dotted line marks the median survival. Whereas control and short-repeat-expressing flies had a similar median survival of 47 and 40.5 days, respectively [GFP, *n*=40 and CTG(20)×, *n*=50], long-CUG-expressing flies lived a median of only 25 days [CTG(250)×, *n*=45). Differences in survival curves were highly significant (*P*<0.0001, log-rank test). (B,C) Heart period mean (HP, B), and arrhythmia index (AI, C) from flies expressing long and short CUG repeats and two types of control flies (F1 from crosses between the *GMH5-Gal4* driver and *w^−^* or *UAS-GFP*). Both parameters are significantly increased in flies expressing long repeats. The bars on the graph show mean values and their standard errors. **P<0.01, ***P<0.001, ns, not significant. (D-G) Representative M-modes (20 s) (D,F), with their respective histogram showing the percentage of beats and their duration (E,G), taken from movies of semi-intact fly hearts expressing long (D,E) or short (F,G) repeats. Red and blue horizontal lines represent the diastolic interval (DI) duration of CUG(250)×- and CUG(20)×-expressing flies, respectively. The systolic interval (SI) and the heart period (HP) length are also indicated in D. The HP histograms, plotted as individual data points (*n*=21, E; *n*=29, G), illustrate the variability of the HP within a group of flies expressing long (E) and short (G) repeats.

Population studies have reported higher mortality and morbidity rates, and a positive correlation between age at onset of DM1 and age at death in affected individuals (Breton and Mathieu, 2009). Similarly, we found that, as a result of long-CUG-repeat expression in heart, median survival and lifespan of flies were reduced at 29°C. The analysis of the survival curves showed that expression of long CUG repeats caused a significant reduction in the median survival of flies. From 47 days in control (*GMH5-Gal4 UAS-GFP*) and 41 days in short-repeat-expressing [*GMH5-Gal4* UAS-CUG(20)×] flies, median survival was reduced to only 25 days in flies expressing long CUG repeats [*GMH5-Gal* UAS-CUG(250)×] (Fig. 2A). Of note, lifespan and median survival of short-repeat-expressing flies was not significantly reduced in comparison to controls (Fig. S3).

To study heart function, adult fly hearts dissected in artificial hemolymph were recorded with a digital video camera. Because previous studies have reported that heart function changes with age, we selected 1-week-old flies for this study. Cardiac contractions were analyzed using a semi-automatic optical heartbeat analysis (SOHA) method to quantify the fly heart functional parameters. M-mode traces of movie clips provided details of the heart wall edge positions (*y*-axis) over time (*x*-axis), illustrating the rhythmicity and the dynamics of the heart contractions (Ocorr et al., 2007).

For the characterization of the cardiac phenotype of long-CUG-expressing flies, we compared their dynamic parameters with short-repeat-expressing flies to reveal the effect of the repeat length, and to two different controls: (1) F1 flies from crossing *GMH5-Gal4* and *w^−^* flies (abbreviated *w^−^*) to take into account potential contributions of the driver to the phenotype; and (2) F1 flies from crossing *GMH5-Gal4* to *UAS-GFP* flies (abbreviated GFP), accounting for the dose of the *UAS* transgenes.

The exposed and largely denervated heart in control and short-repeat-expressing flies showed rhythmic contractions; however, the contractions were clearly arrhythmic in hearts from flies expressing long repeats. We also observed morphological constrictions in small regions along the heart tube where no relaxation phase was observed (see Movie 1). Quantification of the heart period length (HP, defined as the diastolic plus systolic interval) showed that long-repeat-expressing flies exhibited a significantly increased HP compared to control or short-repeat-expressing flies (Fig. 2B). The distribution of all of the measured HP for all flies of a specific genotype was represented in a histogram format, which revealed that the HPs clustered relatively tightly in the short-repeat flies, and that this distribution broadened in long-CUG-expressing flies, emphasizing the increased variability in the HP (compare Fig. 2D,E and 2F,G). The variability in the heart periodicity can be quantified as an ‘arrhythmia index’ (AI) obtained by dividing the standard deviation of the HP by its median (Fig. 2C). Flies expressing long CUG repeats showed approximately a 50% increase in AI compared to control and short-repeat-expressing flies.

### Model flies display systolic and diastolic dysfunction, and reduced contractility

**Fig. 3.**
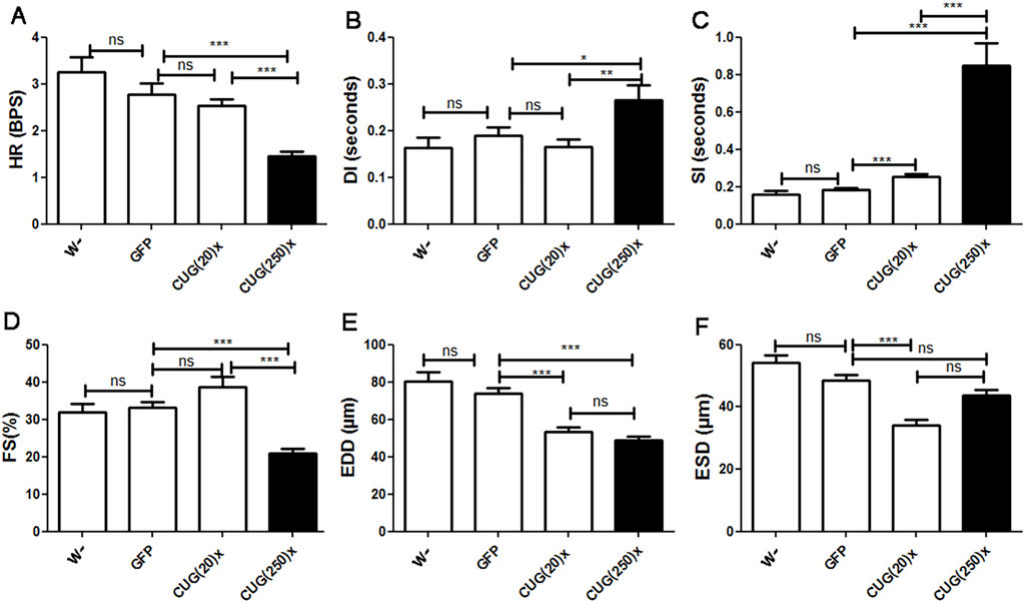
**DM1 model flies displayed systolic and diastolic dysfunction, decreased diastolic diameter and contractility defects.** Flies expressing long CUG repeats showed reduced heart rate [HR, A; expressed in beats per second (BPS)], increased diastolic (DI, B) and systolic (SI, C) intervals, reduced fractional shortening (FS, D), and decreased end diastolic diameter (EDD, E). The reduced contractility did not affect the end systolic diameter (ESD, F), which was not significantly different from controls. Short-repeat-expressing flies displayed an increased SI in comparison to control flies and had a reduced ESD and EDD, without any alteration of FS, suggesting that they have no contractility alteration. Graph bars show mean values and their standard errors (*n* used was between 14 and 29). *P<0.05, **P<0.01, ***P<0.001, ns, not significant.

Alterations of systolic and diastolic function, as well as decreased ventricular ejection fraction, have previously been reported in individuals with DM1 (Dello Russo et al., 2006). To test similar alterations in model flies, we measured the heart rate (HR), the diastolic and systolic intervals (DI and SI, respectively), and the end-diastolic and end-systolic diameters (EDD and ESD, respectively), and calculated the resulting percentage of fractional shortening (% FS), and compared them to control and short-repeat-expressing flies. We found that the increased mean HP, and the correspondingly reduced HR (HP=1/HR) observed in flies expressing long CUG repeats, were caused by systolic and diastolic dysfunction, because both SI and DI (contraction and relaxation period, respectively) were significantly prolonged in comparison to control and short-repeat-expressing flies. To note, the HP of CUG(20)× flies (Fig. 2B) was not significantly different to controls because the slight increment in SI observed was compensated by a decreased DI (Fig. 3B,C). Image analysis of heart contractions also provided cardiac chamber parameters, including EDD and ESD. In addition, the proportional decrease in heart wall diameter during contraction provides an indication of the cardiac output. Control flies displayed an average EDD of about 70 μm and an ESD of 50 μm, and the average FS was higher than 30%. In long-repeat-expressing flies, we observed a significant decrease in EDD (to 50 μm), and also a reduced FS of only 20% (Fig. 3D-F). These data revealed that heart tube volume is reduced and there is a dysfunction of the contractile properties in hearts expressing long CUG repeats. Interestingly, flies expressing short CUG repeats showed both reduced ESD and EDD but normal FS, suggesting that contractile dysfunction resulting in a reduced cardiac output was exclusive to long-repeat-expressing hearts.

### Pentamidine rescues survival, rhythmicity and contractility in the heart-dysfunction model

**Fig. 4.**
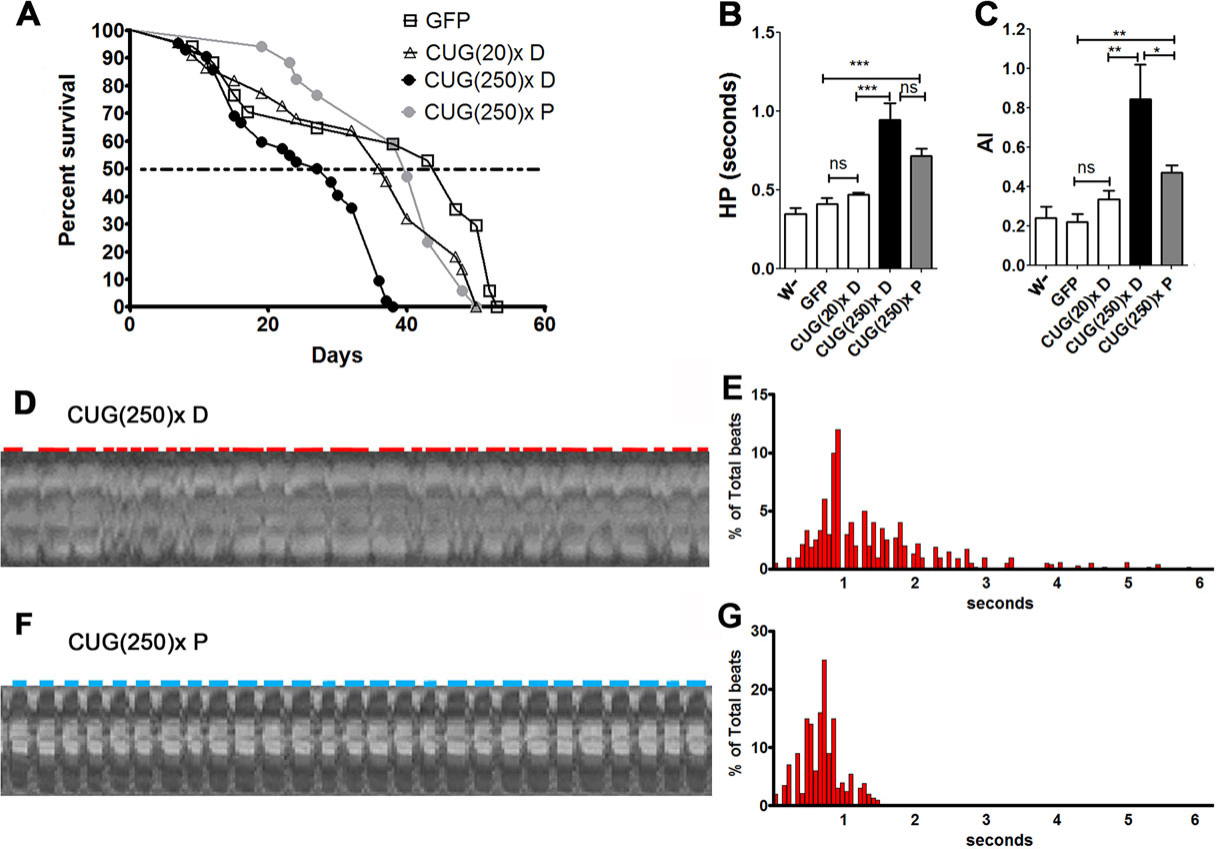
**Pentamidine rescued lifespan, median survival and arrhythmicity in DM1 model flies.** (A) The average percentage of live flies, with the genotypes indicated, versus age (in days). Horizontal dotted line marks median survival. Model flies taking pentamidine [CUG(250)× P] had 40 days of median life, in comparison to only 28 days for long (CUG)250×-expressing flies fed with DMSO [CUG(250)× D]. The survival curves of model flies fed with pentamidine and control flies expressing short repeats fed with DMSO [CUG(20)× D] were not statistically different. (B) The heart period (HP) mean was not significantly altered by pentamidine administration, although a clear trend towards reduction was observed, whereas the arrhythmia index (AI) was strongly reduced (C). Graph bars show the mean values and their standard errors. *P<0.05, **P<0.01, ***P<0.001, ns, not significant. (D-G) Representative M-modes (20 s) (D,F) with their corresponding histograms (E, *n*=25; G, *n*=30) of percentage of beats of a given duration taken from movies of semi-intact flies expressing long repeats fed either with DMSO (D,E) or with pentamidine (F,G). Red and blue horizontal lines denote the diastolic interval (DI).

**Fig. 5.**
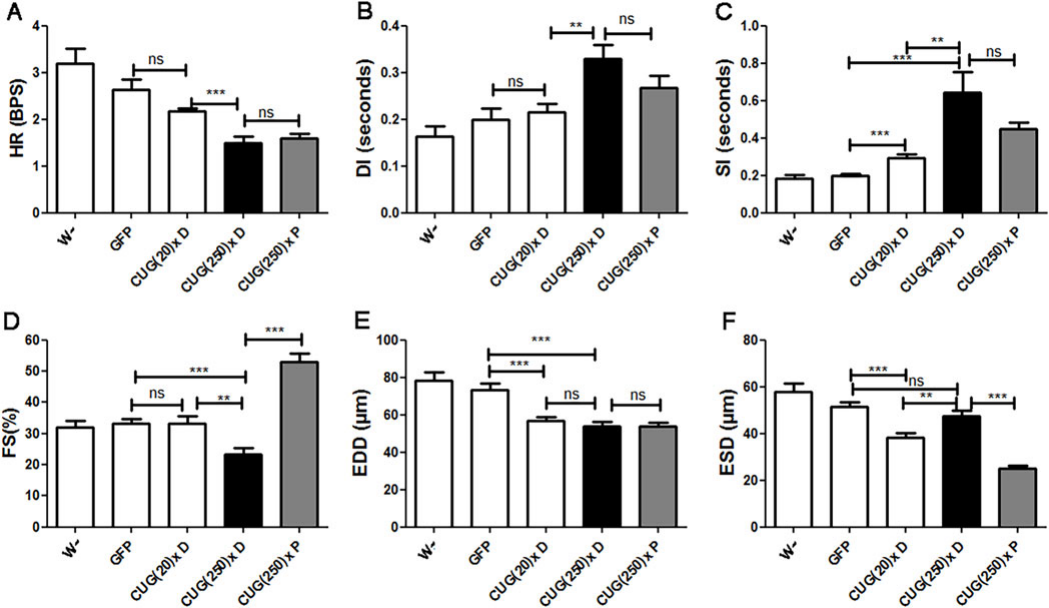
**Pentamidine improved contractility in model flies.** Cardiac function parameters of short- and long-CUG-expressing flies fed either with DMSO [CUG(20)× D and CUG(250)× D] or pentamidine [CUG(250)× P], in comparison to controls. Pentamidine did not modify heart rate (HR, A), diastolic interval (DI, B), systolic interval (SI, C) nor end diastolic diameter (EDD, E) in model flies but strongly reduced end systolic diameter (ESD, F) in these flies, resulting in a relevant increase of fractional shortening (FS, D). Graph bars show the mean values and their standard errors (*n* used was between 14 and 30). **P<0.01, ***P<0.001, ns, not significant.

To assess whether model flies could be used as an *in vivo* tool to search for potential therapeutic compounds against cardiac dysfunction in DM1, we tested the effect of a known anti-DM1 compound on the *Drosophila* heart phenotype. Small molecules designed to inhibit the toxic MBNL1-CUG repeat interaction had shown relevant anti-DM1 activity (Wong et al., 2014). Concretely, pentamidine significantly reduces the formation of ribonuclear foci, and releases MBNL1 from the foci in treated cells. Furthermore, pentamidine has been found to partially rescue the splicing defects of two pre-mRNAs in mice expressing expanded CUG repeats (Warf et al., 2009). To test the effect of this compound in model flies, we added pentamidine diluted in dimethyl sulfoxide (DMSO) to the nutritive media to a final concentration of 1 µM. We tested the effect of DMSO in heart performance prior to these experiments and confirmed that, at the concentration used, it does not alter the cardiac parameters of *Drosophila* (Fig. S4). In comparison to model flies fed with DMSO, which have a median survival of 28 days, the median survival of model flies fed with pentamidine increased up to 40 days, which comes very close to the mean 47-day survival of control flies (Fig. 4A). Moreover, there was a significant reduction in arrhythmicity (see Movies 1, 2 and Fig. 4C). Although in long-repeat-expressing flies fed with pentamidine [CUG(250)× P] mean HP was not significantly reduced (Fig. 4B), there was a clear reduction in the deviation of the HP values, which reflected in a more constrained and grouped HP histogram pattern in comparison to the long-CUG-expressing flies taking DMSO [CUG(250)× D] (compare Fig. 4D,E and F,G). The altered HR, SI and DI detected in the model flies, reminiscent of the systolic and diastolic dysfunction reported in affected humans, were not rescued by pentamidine, although we did observe a conspicuous trend towards normal parameters (Fig. 5A-C). An important recovery of heart contractile properties was observed in pentamidine-treated flies. We observed a decreased ESD and unchanged EDD, resulting into an increased FS (Fig. 5D-F).

### Pentamidine reduces foci and releases Muscleblind in cardiomyocytes of flies expressing long CUG repeats

**Fig. 6.**
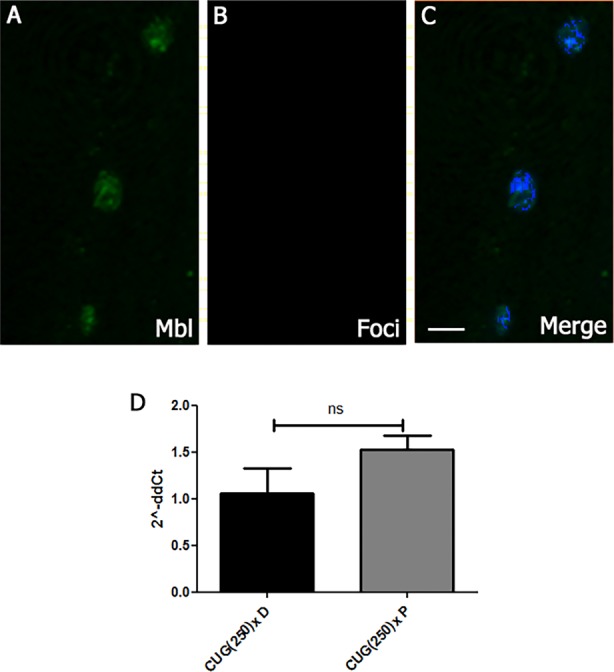
**Pentamidine mechanism of action.** (A-C) Representative fluorescent confocal images of adult heart cells from long-CUG-expressing flies fed with pentamidine. Combined immunostaining of Muscleblind and CUG RNA FISH showed Muscleblind release (A) and no detectable foci (B) in the nucleus of cardiomyocytes of these flies. (C) Merge of A, B and DAPI; counterstaining of nuclei shows dispersed Mbl localization in nuclei. (D) Graph bar represents average fold changes of CUG(250)× expression in logarithmic scale, calculated by the 2^−ΔΔCt^ method, and their confidence intervals. Pentamidine did not significantly alter expression level of CUG repeat RNA in model flies. ns, not significant. Scale bar: 10 µm.

In order to address the mechanism of action of pentamidine, we performed fluorescence in situ hybridization (FISH) and immunofluorescence to detect foci and Muscleblind, in hearts of long-CUG-expressing flies that were fed 1 µM pentamidine. As previously reported in DM1 cells in culture (Warf et al., 2009), ribonuclear foci were absent in cardiomyocyte nuclei and Muscleblind was distributed throughout the nucleus (Fig. 6A-C). Moreover, because biochemical experiments and cell and mouse model studies suggest that pentamidine and related compounds might bind the CTG.CAG repeat DNA and inhibit transcription (Coonrod et al., 2013), we measured expression levels of CUG^exp^ RNA in model flies fed with pentamidine or DMSO, detecting no significant difference (Fig. 6D). These data confirmed that the rescue of the cardiac-dysfunction phenotype achieved by pentamidine was mediated by releasing Muscleblind sequestration rather than reducing toxic RNA expression level.

## DISCUSSION

Here we described, for the first time, the characterization of the cardiac phenotypes of flies expressing either long or short CUG repeats as a DM1 heart-dysfunction model. We measured changes in the heart dynamic parameters, including heart rate, rhythmicity, systolic and diastolic diameters and intervals (ESD, EDD, SI and DI), and FS. Moreover, we show that pentamidine, a compound with previously reported anti-DM1 activity, has the ability to modify the reported cardiac disease phenotypes.

The relevance of the model is supported by the similarities between the cardiac phenotype in DM1 model flies and those documented in individuals with DM1. First, we observed a reduction in the median survival in model flies, which correlates with reports for affected humans (Petri et al., 2012). Cardiac mortality in individuals with DM1 usually occurs because of progressive left ventricular dysfunction, ischemic heart disease, pulmonary embolism, or as a result of unexpected sudden death (SD) associated with the corrected prolonged QT interval (period including electrical depolarization and repolarization of the ventricles) (Park et al., 2013). Second, heart conduction abnormalities are common in individuals with DM1 (Groh et al., 2008; McNally and Sparano, 2011). Similarly, we observed a significantly increased HP and arrhythmia index in our fly model. Although several arrhythmias have been reported in individuals with DM1, recent studies found that atrial fibrillation (AF) and atrial flutter (AFL) are frequent in DM1 and are linked to increased mortality (Brembilla-Perrot et al., 2014). Thirdly, the altered SI and DI observed in DM1 flies are reminiscent of systolic and diastolic dysfunction reported in humans with the disease (Penisson-Besnier et al., 2008; Hermans et al., 2012). Recently, echocardiography-Doppler found an increase of the mean left-atrial diameter and an increase of the mitral deceleration time in DM1 individuals, suggesting diastolic abnormalities (Fayssoil et al., 2014). Left ventricular systolic dysfunction (LVSD) has also been reported in 7.2% of affected individuals (Petri et al., 2012). Moreover, individuals with DM1 with cardiac abnormalities generally show various extents of heart chamber dilation and hypertrophy, which results in a decreased ventricular ejection fraction (Dhand et al., 2013; Chaudhry and Frishman, 2012; McNally and Sparano, 2011; Pelargonio et al., 2002; Hermans et al., 2012) and correlates with the reduced FS we observed in model flies.

We have also found cardiac defects in flies expressing short CUG repeats; mainly, slightly increased SI and reduced cardiac tube diameters. Interestingly, model mice acutely overexpressing a normal-length *DMPK* 3′ UTR mRNA reproduced cardinal features of myotonic dystrophy, including myotonia, cardiac conduction abnormalities, histopathology and RNA splicing defects in the absence of detectable nuclear inclusions (Storbeck et al., 2004; O'Cochlain et al., 2004; Mahadevan et al., 2006). Authors hypothesized that the effects of overexpressing many *DMPK* 3′ UTR transcripts with a small repeat number might be pathogenically equivalent to expressing mutant transcripts with hundreds of CUGs. In our flies, we measured a similar level of expression of the repeats in short- and long-expressing flies but used a potent promoter for overexpression (*GMH5-Gal4*). This driver includes a *UAS-GAL4* element, allowing strong and continuous expression after induction. According to our data, controlled by this driver, expression of 20 CTG repeats does cause the formation of ribonuclear foci retaining Muscleblind but is enough to induce some cardiac phenotypes. Because MBNL loss in mice has been recently proven to be enough to cause cardiac pathology (Dixon et al., 2015), the finding of cardiac pathology in the absence of Muscleblind sequestration in our model flies becomes highly relevant because it shows a cardiac dysfunction mechanism that might be Muscleblind-independent in CUG-expressing flies.

The rescue of the cardiac parameters using pentamidine supports the specificity of the heart-dysfunction phenotype and confirms the therapeutic effect of pentamidine in an *in vivo* model. Interestingly, pentamidine did not completely re-establish all cardiac parameters. Diastolic and systolic function remained altered, suggesting that either the effect of the pentamidine is limited, or the defect itself is not susceptible to therapeutic recovery in adults. This could be the case for alterations occurring early in development because the *Drosophila* heart is one of a few structures that persist during pupal morphogenesis, although it undergoes extensive remodeling (Rizki and Rizki, 1978). Similarly, in humans, CUG RNA toxicity during development could cause alterations in heart physiology or anatomy that cannot be modified by treatment in adults. This situation is not reproduced by inducible models expressing the CUG expansions after birth, only in models with constitutive CUG-repeat expression. Another heart-dysfunction feature that is not rescued by pentamidine is the reduced EDD found in the model flies. Of note, EDD was equally reduced in both flies expressing short and long repeats, suggesting that it might not require Muscleblind sequestration. Therefore, pentamidine might not be able to modify this parameter. Importantly, the rescue achieved by pentamidine was enough to increase FS, which correlates with hemolymph volume ejected, and corrected the heart arrhythmia, both of which are thought to be the most prevalent causes of sudden death in individuals with DM1.

The long-repeat-expressing flies recapitulate many of the pathological and molecular features of DM1, including reduced survival, arrhythmias, systolic and diastolic dysfunction, and Muscleblind retention into ribonuclear foci. The rescue obtained by pentamidine treatment confirms that the DM1 model described has a sensitized phenotype that is suitable to unravel the mechanism of heart dysfunction in DM1 and to test potential therapeutic approaches in future studies.

## MATERIALS AND METHODS

### *Drosophila* strains

Self-priming (CTG)20× and (CAG)20× synthetic oligonucleotides were cloned into the linearized pUAST vector to generate pUAST-CTG(20)×. PUAST-CTG(250)× was constructed by subcloning 500 uninterrupted CTG repeats from the pcDNA-CTG(500)× vector, which was a kind gift from Dr Partha Sarkar (Department of Neurology, University of Texas Medical Branch, TX). After cloning into pUAST and amplification into the STBL3 (Invitrogen) *Escherichia* *coli* strain at 20°C, the 500 pure CTG repeats contracted to 250 CTG units. Transgenic flies carrying 250 or 20 pure repeats were generated by standard P-mediated transgenesis (BestGene Inc., Chino Hills, CA, USA). Transgenic lines carrying both long and short repeats were selected on the basis of moderate transgene expression and reproducibility of the phenotypes studied. In the fly lines used, the transgenes were located by inverse PCR to chromosome 2. The cardiomyocyte-specific driver *GMH5-Gal4* (kindly provided by the laboratory of Dr Rolf Bodmer in the Burnham Institute, CA, USA) is a 900 bp heart enhancer fragment 73 from the *tinman* gene that was cloned into the P{GaWB} vector upstream of the *Gal4* sequences. This driver was enhanced with multiple copies of a *UAS-Gal4* element allowing stronger myocardial expression and a *UAS-GFP* element allowing detection of the expression tissue (Wessells et al., 2004). All fly lines were maintained at 25°C with standard *Drosophila* food and standard day-night cycle.

### Quantification of CUG-repeat expression level

Total RNA was extracted from ten flies per genotype using Trizol reagent (Sigma). DNase I treatment and reverse transcription were performed as previously reported (Llamusi et al., 2013). To quantify the expression of CUG RNA, the common SV40 terminator in the pUAST vector was used as target of the primers (F: 5′-GGAAAGTCCTTGGGGTCTTC-3′, R: 5′-GGAACTGATGAATGGGAGCA-3′). Expression levels were normalized to the reference gene *rp49* (F: 5′-ATGACCATCCGCCCAGCATAC-3′, R: 5′-ATGTGGCGGGTGCGCTTGTTC-3′) using the SYBR^®^ Green mixture (Roche) under 2^−ΔΔCt^ method. For each genotype, three biological samples were used and three technical replicates were performed.

### Detection of CUG-repeat length

To confirm the length of the repeats in the *UAS*-CTG(20)× and *UAS*-CTG(250)× transgenes, 40 ng of genomic DNA was used as a template for the PCR amplification with KAPA HiFi (BIOSYSTEMS) and the primers: F 5′-GCAACTACTGAAATCTGCCAAGA-3′ and reverse- 5′-GTTGAGAGTCAGCAGTAGCC-3′, which flank the repeats. The region amplified by the primers includes the short repeats (60 bp) and 375 bp of the CTG(20×) plasmid, and the long repeats (750 bp) and 428 bp of the CTG(250)× plasmid. PCR amplification was performed under the following conditions: 95°C for 2 min, followed by 30 cycles of 98°C for 20 s, 65°C for 30 s, 72°C for 1 min and final extension at 72°C for 5 min. The PCR products were analyzed by electrophoresis at 110 V in 1.5% agarose gels.

### Pentamidine treatment

Pentamidine was added to the standard food to a final concentration of 1 µM in 0.1% DMSO (Applichem). The control group of flies was fed with 0.1% DMSO. Flies were transferred every 3 days to new fresh food media, with or without pentamidine for the duration of their whole lifespan in life survival experiments or every 7 days in the case of the group used for cardiac analysis.

### Survival analyses

For survival analyses, a minimum of 40 female flies from the corresponding genotypes were collected and kept at 29°C. Flies were transferred to new fresh nutritive media every second day and scored for deaths daily. Statistical analysis was performed with a log-rank test using the GraphPad Prism5 software.

### Cardiac physiological analysis

For the physiological analysis, female flies were collected just after eclosion and were maintained for 7 days at 29°C. For the heart beat recordings, semi-intact heart preparations were made as previously described (Ocorr et al., 2007; Magny et al., 2013). An inverted Leica DM Irbe microscope, connected to a DFC450C Leica digital camera, was used to take 20 s recordings at 29 frames/s. Different cardiac parameters were measured using Fly_heart_analysis (SOHA) software based on Matlab R2009b (MathWorks, Natick, MA, USA) (Ocorr et al., 2007). For the statistical analysis, Student’s *t*-test was used with Welch's correction when the variances were different.

### Fluorescent immunofluorescence analysis

Fly hearts were dissected from 7-day-old females, fixed for 20 min in 4% paraformaldehyde, and washed in PBT (PBS containing 0.3% Triton X-100). Muscleblind staining and FISH to detect ribonuclear foci were performed as previously described (Llamusi et al., 2013). For double Muscleblind and GFP staining, dissected hearts were washed in PBT and incubated in blocking buffer (PBS containing 0.3% Triton X-100, 5% donkey serum and 0.5% bovine serum albumin) for 30 min prior to overnight incubation at 4°C with primary antibodies sheep-anti-Muscleblind (Houseley et al., 2005) and rabbit anti-GFP (#G10362, Invitrogen) diluted 1/500 in blocking buffer. After several PBT washes, the tissue was incubated for 45 min with biotin-conjugated secondary antibodies (#31840, Thermoscientific) at 1:200 dilution and then incubated with ABC solution (ABC kit, VECTASTAIN) for 30 min at room temperature, followed by washes and 45 min incubation with anti-rabbit FITC (#F9887-5ML, Sigma) secondary antibody and streptavidin–Texas-red (1:1000, #SA5006, VECTOR). For phalloidin staining, phalloidin (#P1951, Sigma) was diluted 1:1000 in PBT and tissues were incubated for 20 min. Samples were mounted in Vectashield (Vector) as described before (Alayari et al., 2009). All confocal images were taken in an Olympus FV1000 microscope.
